# Primary Cutaneous B‐Cell Lymphomas: An Updated Portrait of Classification, Biology, and Clinical Management

**DOI:** 10.1111/ejh.70053

**Published:** 2025-10-28

**Authors:** A. Bernardelli, E. Carazzai, B. Bugnotto, F. Bellinato, M. Krampera, C. Visco

**Affiliations:** ^1^ UOC di Ematologia e CTMO, DAI Medico Generale Azienda Ospedaliera Universitaria Integrata di Verona Verona Italy; ^2^ Hematology and Bone Marrow Transplant Unit, Section of Biomedicine of Innovation, Department of Engineering for Innovative Medicine (DIMI) University of Verona Verona Italy; ^3^ Section of Dermatology and Venereology, Department of Medicine University of Verona Verona Italy

**Keywords:** B‐cell neoplasms, cutaneous B‐cell lymphoma, multidisciplinary management, rare cutaneous lymphomas, targeted therapies

## Abstract

Primary cutaneous B‐cell lymphomas (CBCL) represent a clinically and biologically heterogeneous group of extranodal non‐Hodgkin lymphomas confined to the skin at the time of diagnosis. They account for approximately 25% of all primary cutaneous lymphomas and are subclassified into distinct entities according to the World Health Organization—European Organization of Research and Treatment of Cancer (WHO–EORTC) classification and International Consensus Classification (ICC). including primary cutaneous follicle center lymphoma (PCFCL), primary cutaneous marginal zone lymphoma (PCMZL), primary cutaneous diffuse large B‐cell lymphoma, leg type (PCDLBCL‐LT), intravascular large B‐cell lymphoma (IVLBCL) and Epstein–Barr virus–positive mucocutaneous ulcer (EBVMCU). These subtypes differ significantly in clinical behavior, histopathological features, molecular alterations, and prognosis. Indolent forms such as PCFCL and PCMZL are typically managed with local therapies and are associated with an excellent prognosis. In contrast, aggressive variants such as PCDLBCL‐LT require systemic treatment and are linked to poorer outcomes. EBVMCU, despite its alarming histological appearance, generally follows a benign and self‐limiting course. This review provides an updated overview of the current diagnostic criteria, clinical management strategies, and emerging molecular insights for each CBCL subtype. It also emphasizes the importance of a multidisciplinary approach and discusses the challenges of prognostication, along with the evolving but still limited role of innovative therapies.

## 1 Introduction

Primary cutaneous lymphomas represent a diverse and complex group of extranodal non‐Hodgkin lymphomas affecting the skin, with no evidence of extracutaneous disease at the time of diagnosis. These malignancies are broadly categorized based on the lineage of the neoplastic cells, with the majority arising from T cells. However, approximately 25% of cases are of B‐cell origin and are collectively referred to as primary cutaneous B‐cell lymphomas (CBCL) [1, 2, 3] (Table 1). The incidence of CBCL has been steadily increasing and is currently estimated at approximately four cases per million individuals. Epidemiological data indicate that the highest incidence rates occur in males, individuals from Western populations, and adults over the age of 50 [4]. The classification and understanding of CBCLs have significantly evolved, particularly with the contributions of the 2018 joint World Health Organization (WHO)–European Organization for Research and Treatment of Cancer (EORTC) classification and the more recent International Consensus Classification (ICC). These frameworks have established a standardized approach to diagnosis and nomenclature, recognizing four principal entities within CBCLs: primary cutaneous follicle center lymphoma (PCFCL)—typically presenting as slowly progressive lesions on the scalp, forehead, or trunk, and generally associated with an excellent prognosis; primary cutaneous marginal zone B‐cell lymphoma (PCMZL)—an indolent lymphoma often manifesting as pink to red papules, plaques, or nodules, predominantly on the arms and trunk; primary cutaneous diffuse large B‐cell lymphoma, leg type (PCDLBCL, LT)—a more aggressive subtype that commonly affects the lower extremities and is associated with a less favorable prognosis than other CBCLs. Intravascular large B‐cell lymphoma (IVLBCL) is a rare and aggressive subtype of large B‐cell lymphoma characterized by a unique pattern of intravascular growth, typically without significant lymphadenopathy, and occasionally confined to the skin Epstein–Barr virus–positive mucocutaneous ulcer (EBVMCU)—a newly recognized lymphoproliferative disorder characterized by self‐limited, ulcerative lesions of the skin or mucosa, often occurring in immunosuppressed or elderly individuals [1, 2, 3]. Diagnosing and managing PCBCL require a multidisciplinary approach, involving close collaboration among dermatologists, pathologists, hematologists, and radiotherapists. PCMZL and PCFCL are typically indolent and are commonly treated with local radiotherapy (RT) alone, yielding excellent outcomes. In contrast, the more aggressive DLBCL‐LT is generally managed with systemic chemotherapy combined with involved‐field radiotherapy and is associated with less favorable survival outcomes. This review aims to provide a comprehensive overview of the current understanding of CBCLs, highlighting their clinical, histopathological, and molecular features, to inform accurate diagnosis, appropriate risk stratification, and evidence‐based therapeutic strategies.

**TABLE 1:** Features of primary cutaneous B‐cell lymphomas.

	PCFCL	PCMZL	PCDLBCL‐LT	IVLBCL	EBVMCU
Epidemiology					
Mean age of onset	5–6th decade	5–6th decade	7–8th decade	7th decade	7th decade
Sex predominance	Male	Male	Female	Female	Female
% cases of PCBCL	30%–50% (in Caucasians)	25%–30%	20%–40%	Rare (more often in Caucasians)	Rare
Clinical features	Asymptomatic and localized plaques and nodules	Asymptomatic or slightly pruritic, solitary or multiple clustered plaques and nodules	Solitary or multiple, rapidly growing, multifocal nodules and tumors	Mild to severe symptoms, heterogeneous single or multiple skin lesions	Solitary, well‐circumscribed ulcerative lesion
Frequent sites	Head or trunk	Trunk, arms or head	Lower extremities	Skin, frequent CNS involvement	Skin, oropharyngeal mucosa, or gastrointestinal tract
Histology	Dermal or subcutaneous mixture of centrocytes and large centrocytes infiltrates in follicular, follicular and diffuse or diffuse growth patterns; background fibrosis and sclerosis with stromal reaction; without a normal mantle zone	Polymorphus. Nodular or diffuse dermal infiltrate composed of small to medium‐sized lymphocytes (“centrocyte‐like”), with monocytoid B‐cells, plasma cells, reactive T‐cells; no stromal reaction	Diffuse, dense infiltrate of large atypical lymphoid cells predominantly involving the dermis and frequently extending into the subcutaneous tissue; no stromal reaction	Lymphoma cells in vessel lumina with different growth patterns: discohesive; cohesive; marginating pattern	Dense polymorphic inflammatory infiltrate, with transformed B cells resembling Hodgkin/Reed–Sternberg cells. Consistently positive for Epstein–Barr virus‐encoded RNA
Immunohistochemical (IHC)	CD20+, BCL6+, CD21+, CD43+, monotypic Igs, CD10‐ (< 25% +), BCL2‐(weak+), MUM1/IRF4 —	CD20+, CD79a+, PAX5+, BCL2+, CD5‐, CD10‐, BCL6—	CD20+, CD79a+, BCL6+/−, PAX5+, IgM+, BCL2+, MUM1/IRF4+, CD10—	CD20+, CD79a+, PAX5+, BCL2+, MUM1/IRF4+, CD5+/−, PDL1+, CD10‐, BCL6‐, CD30‐, IgM+	CD20+/−, PAX5+/−, IRF4/MUM1+, CD10‐, BCL6+, CD30+, CD15+/−, EBER+
Molecular and cytogenetic findings					
*BCL2* rearrangement/t(14;18)	Negative (10%–40% positive)	Negative	Negative	Negative	Negative
BCL6 or MYC rearrangement	Negative	Negative	Positive (30% BCL6, 30% MYC)	Rare MYC rearrangement	Negative
Other	< 10% of cases mutation in epigenetic modifiers	t(14;18)(q32,q21) (27%), FAS mutations (60%)	*MYD88* (60%), *CD79B* (20%)	MYD88 L265P (44%); CD79B (26%)	—

## 2 Primary Cutaneous Follicle Center Lymphoma (PCFCL)

PCFCL represents the most common type of PCBCL in Caucasians, accounting for up to 30%–50% of all PCBCL [5]. In contrast, it is the least common PCBCL subtype in Asian countries, such as Japan and Korea [6]. It is considered a separate entity in the WHO‐EORTC classification of primary cutaneous lymphomas as well as in the new WHO classification of hematopoietic and lymphoid tissue tumors [7].

The median age at onset is between the 5th and 6th decades, with a male predominance.

### 2.1 Clinical Features

**FIGURE 1:**
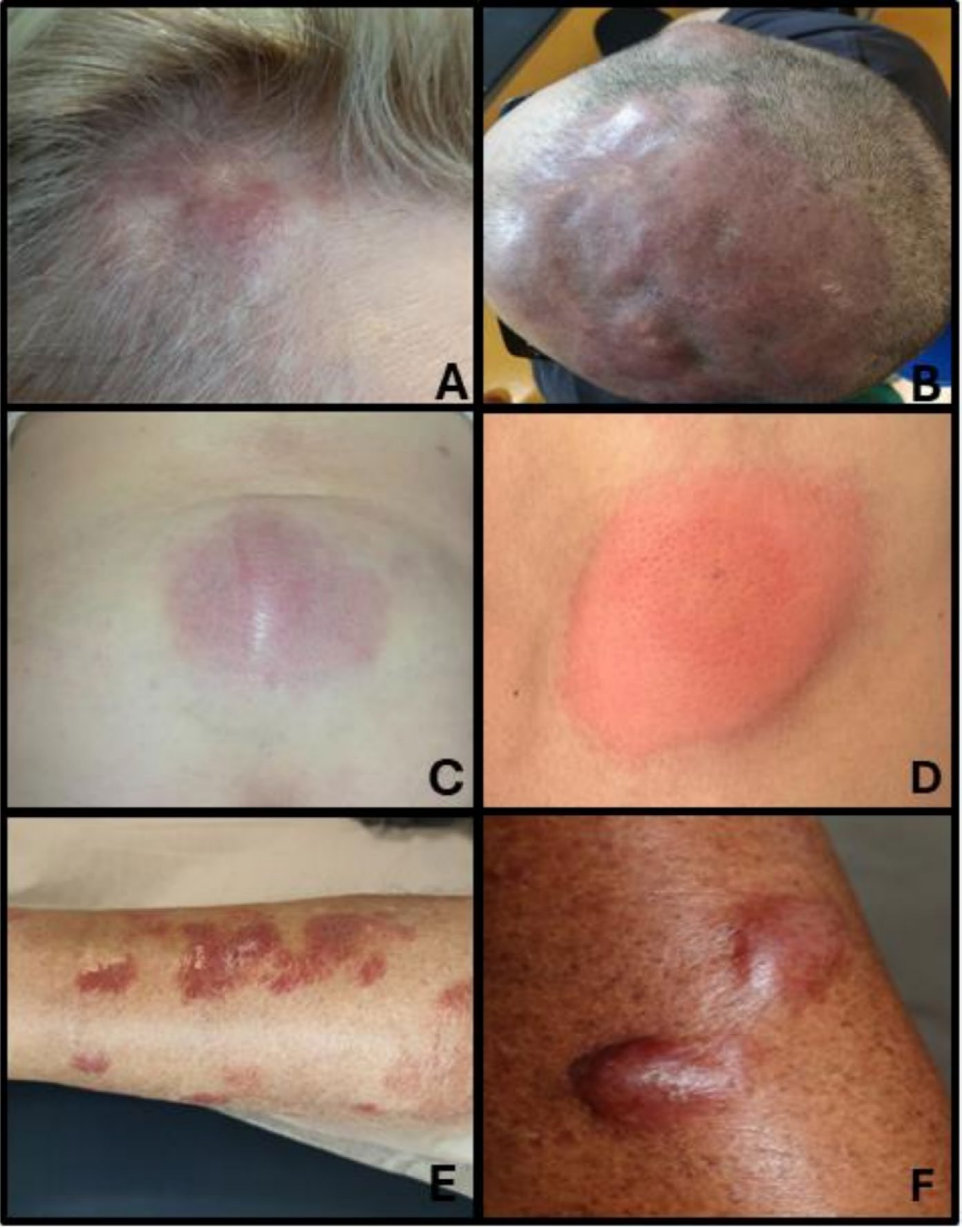
(A) and (B) Primary cutaneous follicle center lymphoma, (C) primary cutaneous marginal zone lymphoma, (D) insect bite lesion, (E) and (F) primary cutaneous diffuse large B‐cell lymphoma, leg type.

PCFCL typically presents as solitary red to violaceous plaques, nodules or tumors with a smooth, shiny, mamillated surface that can be single or grouped. These lesions are most frequently located on the head (Figure 1A,B), neck, or trunk and rarely affect the legs. Erythematous papules and slightly indurated plaques surrounding the tumor are characteristic findings. Multifocal skin involvement is uncommon (10%–15% of cases) and lacks prognostic significance. In the absence of treatment, lesions usually show a slow, progressive increase in size (most often lesions don't exceed approximately 3 cm in diameter), while dissemination to extracutaneous sites is rare [8]. Most patients are asymptomatic or present only with localized pruritus. Ulceration is rare. Atypical manifestations have been described in the literature and may mimic facial dermatoses such as lupus tumidus, granulomatous rosacea, lupus miliaris disseminates faciei, B‐cell pseudolymphoma, IgG4 related disease but also solid organ metastasis [9]. Scalp involvement may lead to alopecia. A clinical variant, historically known as “Crosti's lymphoma” or “reticulohistiocytoma of the dorsum”, is characterized by figurate, annular, concentric plaques with peripheral macules or papules on the trunk [10]. Extracutaneous involvement is uncommon. These presentations can pose a diagnostic challenge, as they may be difficult to distinguish from inflammatory lesions, exaggerated reactions to arthropod bites (Figure 1D), lupus tumidus, other cutaneous neoplasms or non‐B‐cell cutaneous lymphoma.

### 2.2 Histopathology and Biological Features

Histological examination reveals a bottom‐heavy lymphoid infiltrate in the deep dermis and/or subcutaneous tissue, displaying a prominent follicular pattern. The follicles show a variable composition of centrocytes and centroblasts and typically lack a well‐defined mantle zone. The architectural pattern varies along a continuum, including follicular, nodular, diffuse growth patterns, or a combination thereof. Surrounding the follicles are small lymphocytes and histiocytes, admixed with inflammatory cells such as eosinophils and plasma cells [11, 12]. The neoplastic follicles are composed of B cells (CD20+, CD79a+) that express follicle center‐associated markers such as BCL‐6 and are embedded within a network of follicular dendritic cells highlighted by CD21 or CD35. These cells typically show monotypic surface immunoglobulin expression, variable CD43 positivity, and reduced or absent CD10 expression [13, 14]. CD10 is more frequently expressed in PCFCL with a follicular growth pattern. Most cases are negative for MUM1/IRF4 and FOXP1, distinguishing them from other entities as PCDLBCL‐LT [15]. However, since Bcl‐2, MUM‐1, and to a lesser extent FOXP1 are also expressed by a small minority of PCFCL, these markers cannot be used as a golden standard to differentiate between both conditions. In contrast to nodal follicular lymphomas, PCFCLs often lack BCL‐2 expression and rarely harbor the t(14;18)(q32;q21) translocation involving the BCL2 gene [16, 17]. Recurrent mutations in the epigenetic modifiers CREBBP, KMT2D, EZH2, and EP300 have been frequently observed in secondary cutaneous follicular lymphoma but are rarely found in PCFCL. In contrast, TNFRSF14 mutations are commonly detected in PCFCL [18].

### 2.3 Diagnosis and Staging

The diagnosis is established based on histopathological features, integrated with clinical correlation, immunohistochemical profiling, immunoglobulin heavy chain clonality analysis, and exclusion of differential diagnoses. A definitive diagnosis requires a representative biopsy of sufficient length and diameter. An excisional biopsy is preferable; however, the diameter should be at least 4 mm whenever possible in the case of punch biopsies. The histological differentiation between PCFCL exhibiting a diffuse growth pattern and PCDLBCL‐LT may present a significant diagnostic challenge. This is particularly true for PCFCL variants containing numerous centroblasts, which can closely mimic the high‐grade morphology of PCDLBCL‐LT. Accurate distinction relies on a combination of architectural and immunophenotypic findings. PCDLBCL‐LT typically consists of confluent sheets of centroblasts and immunoblasts and exhibits a characteristic phenotype: strong and uniform BCL2 expression, positivity for IgM and MUM1/IRF4, and a nongerminal center, activated B‐cell profile. In contrast, PCFCL often demonstrates a vaguely nodular architecture with a partially preserved CD21+/CD23+ follicular dendritic cell meshwork and a prominent reactive T‐cell infiltrate. Immunohistochemically, it typically shows weak or absent BCL2 expression, negativity for IgM and MUM1, and a germinal center B‐cell phenotype [1, 19].

Staging is essential to exclude the presence of extracutaneous disease. It should include history and the patient's examination, appropriate laboratory tests (including comprehensive serum chemistries, serum lactate dehydrogenase, and serum protein electrophoresis), and appropriate imaging studies of the chest, abdomen, and pelvis (CT scan and/or FDG‐PET). Bone marrow biopsy could be considered in cases positive for BCL‐2, CD10, or t (14, 18) translocation at presentation [20, 21]. PCFCLs are generally negative for t(14;18) translocation, but when present it may have prognostic implications. This genetic alteration, although uncommon, has been associated with an increased risk of cutaneous relapse and a higher probability of extracutaneous dissemination. Moreover, the detection of t(14;18) may also raise the suspicion of a cutaneous manifestation of systemic follicular lymphoma, underscoring the need for careful staging [15, 20].

The staging system, recommended by the ISCL/EORTC, is intended solely for anatomical documentation of disease extent, following the TNM classification (Table 2), likewise other B‐cell cutaneous lymphomas. However, staging in cutaneous B‐cell lymphomas has limited prognostic value, because histopathology has the major role in risk‐stratification [22].

**TABLE 2:** TNM classification for cutaneous lymphomas, proposed by the ISCL and the cutaneous lymphoma task force of the EORTC.

Staging		
T (tumor)	1	Single cutaneous lesion a: <5 cm b: > 5 cm
	2	Regional skin involvement (multiple lesions limited to one or two contiguous regions) a: all lesions, diameter < 15 cm b: all lesions, diameter 15–30 cm c: all lesions, diameter > 30 cm
	3	Generalized skin involvement a: involving two noncontinuous regions b: involving ≥ 3 regions
N (nodules)	0	No clinical or pathologic LN involvement
	1	Involvement of one peripheral LN region that drains an area of current or prior skin involvement
	2	Involvement of two or more peripheral LN or involvement of any LN region that does not drain an area of current or prior skin involvement
	3	Involvement of central LN
M (metastasis)	0	No extracutaneous non‐LN disease
	1	Evidence of extracutaneous non‐LN disease

*Note:* Definition of body regions (limits): Head and neck (inferior border—superior border of clavicles, T1 spinous process); Chest (superior border—superior border of clavicles; inferior border—inferior margin of rib cage; lateral borders—mid‐axillary lines, gleno‐humeral joints, inclusive of axillae). Abdomen/genital (superior border—inferior margin of rib cage; inferior border—inguinal folds, anterior perineum; lateral borders—mid‐axillary lines). Upper back (superior border—T1 spinous process; inferior border—inferior margin of rib cage; lateral borders—mid‐axillary lines). Lower back/buttocks (superior border—inferior margin of rib cage; inferior border—inferior gluteal fold, anterior perineum, inclusive of perineum; lateral borders—mid‐axillary lines). Each upper arm (superior borders—glenohumeral joints, exclusive of axillae; inferior borders—ulnar/radial‐humeral/elbow joint). Each lower arm/hand (superior borders—ulnar/radial‐humeral/elbow joint). Each upper leg and thigh (superior borders—inguinal folds, inferior gluteal folds; inferior borders—mid‐patellae, mid‐popliteal fossae). Each lower leg, foot (superior borders—mid‐patellae, mid‐popliteal fossae) [21].

### 2.4 Treatment

PCFCL is an indolent lymphoma with a disease‐related 5‐year survival of 95% [23]. When clinically appropriate, a conservative therapeutic approach should always be prioritized. Patients presenting with a solitary/localized lesion benefit from treating each lesion with curative intent. First‐line treatment consists of involved site radiotherapy (ISRT), low‐dose radiotherapy or surgical excision, with a complete remission rate approaching 100% [24, 25]. There is no consensus on the optimal curative radiation dose for indolent PCFCL. Recommended doses range from 24 to 40–45 Gy, with a margin of clinically uninvolved skin of at least 1–1.5 cm; the NCCN and ILROG suggest a dose between 24 and 30 Gy [26, 27]. Low doses of RT (2–4 Gy in 2 fractions) achieve a complete remission rate of 72%, with 30% of lesions requiring retreatment [28]. Surgery appears to be an equally effective treatment option but is typically reserved for select cases where small lesions may be removed with minimal and nondisfiguring surgery. No details are provided concerning excision margins [29, 30]. Topical therapies with high‐potency steroids, imiquimod, nitrogen mustard, and bexarotene may be effective to treat symptomatic lesions [31]. Patients with few scattered lesions may be treated with both ISRT of all visible skin lesions as well as a wait‐and‐see policy associated with the treatment of only symptomatic skin lesions. Rarely, patients with PCFCL may show a locally aggressive course and some have suggested the possibility of transformation to DLBCL, suggesting that “watch and wait” patients require close clinical follow‐up [32]. Intralesional rituximab is highly effective with a rate of complete response of 60%–80% but is unsuitable due to the need for multiple injections, associated pain, and mild adverse events. In cases of systemic disease, first‐line treatment typically consists of systemic rituximab (375 mg/m^2^ weekly for 4–8 weeks), followed, when appropriate, by combined modalities such as involved‐site radiotherapy (ISRT) or surgical excision to enhance local disease control [33]. In PCFCL cases presenting with diffuse extracutaneous involvement, extensive cutaneous dissemination, or lesions affecting the lower extremities, systemic treatment with rituximab may be considered. The addition of multi‐agent chemotherapy, such as R‐CHOP (rituximab, cyclophosphamide, doxorubicin, vincristine, and prednisone), along with involved‐site radiotherapy (ISRT), can be used in selected high‐risk cases. However, polychemotherapy should be reserved for very carefully selected patients due to its associated toxicity. A systematic review reported complete response (CR) rates of 85% with multi‐agent chemotherapy, although relapse occurred in approximately 44% of cases [22, 34].

Follow‐up recommendations should be individualized based on the clinical situation. Generally, guidelines recommend a follow‐up every 6 months, with a complete cutaneous and nodal examination. Additional testing, such as histology, blood examination, and imaging should be performed only when clinically indicated. Recurrence occurs in 20%–50% of cases, but it is usually confined to the skin and does not impact overall prognosis. Although most relapses occur within the first four years, a persistent risk of recurrence (ranging from 2% to 6%) has been observed even beyond 10 years [35].

## 3 Primary Cutaneous Marginal Zone Lymphoma (PCMZL)

PCMZL is an indolent B‐cell lymphoma that accounts for 7%–9% of all primary cutaneous lymphomas and 25%–30% of CBCL [35, 36]. The 2022 WHO Classification and the 2022 ICC acknowledge PCMZL as a distinct entity, separate from other mucosa‐associated lymphoid tissue (MALT) lymphomas, based on its unique clinical presentation and biological behavior. While the WHO maintains its classification of PCMZL as an indolent lymphoma, the ICC 2022 underscores its markedly indolent course and reclassifies it as a primary cutaneous marginal zone lymphoproliferative disorder (PCMZL‐LPD), highlighting its low‐grade nature and favorable prognosis [2, 3].

### 3.1 Clinical Features

PCMZL‐LPD predominantly affects adult male patients, with a median age in the fifth to sixth decade of life. Although rare during childhood, it represents the most common cutaneous B‐cell lymphoma seen in children and adolescents [37]. Patients with PCMZL‐LPD are reported to have 99%–100% 5‐year disease‐specific survival and 93% OS at 5 years. Only 4% of patients developed extracutaneous disease [38]. *Borrelia burgdorferi* infection has been associated with skin MZL in some cases in Europe. However, it has not been observed in the U.S., Asia, and some parts of Europe, thereby challenging its proposed aetiological role [39]. Furthermore, PCMZL‐LPD is significantly associated with systemic conditions, including gastrointestinal disorders, autoimmune diseases, and other malignancies [34].

Clinically, it typically presents as asymptomatic or mildly pruritic solitary or grouped papules, plaques, or nodules, most located on the trunk (Figure 1C) or upper extremities. Involvement of the head and neck region is observed in approximately one‐third of cases, while leg involvement is relatively uncommon [38, 40]. Systemic symptoms are usually absent. In some cases, spontaneous regression of the lesions may be observed [36]. Ulceration is uncommon. Anetoderma can be seen, with loss of elastic tissue in the dermis [41]. PCMZL‐LPD can arise in skin affected by acrodermatitis chronica atrophicans as a consequence of Borrelia infection. The diagnosis of PCMZL can be challenging, as it often shares histopathological features with reactive cutaneous lymphoid hyperplasia; demonstration of light chain restriction or clonal IGH rearrangements, together with clinicopathological correlation, may facilitate the diagnosis.

### 3.2 Histopathology and Biological Features

PCMZL‐LPD typically presents as nodular or diffuse dermal infiltrate composed of small to medium‐sized lymphocytes, occasionally extending into subcutis and subcutaneous fat, while the epidermis is typically unaffected. The infiltrate consists of small lymphocytes, marginal zone B‐cells (centrocyte‐like cells), lymphoplasmacytoid cells and plasma cells, admixed with small numbers of centroblast‐ or immunoblast‐like cells and many reactive T‐cells. A predominance of T cells is present in many cases, with cytological atypia, irregular nuclear contours, and pale cytoplasm [38]. Reactive germinal centers are commonly observed and may be surrounded by a population of small or medium‐sized cells with irregular nuclei, inconspicuous nucleoli, and abundant pale cytoplasm (marginal zone B‐cells). Monotypic plasma cells are frequently found at the periphery of the lymphoid nodules and in the superficial dermis just beneath the epidermis. Small lymphocytes and large blasts are also commonly observed. Eosinophils are present in about 25% of cases [42].

Neoplastic B‐cells in PCMZL‐LPD express markers such as CD20, CD79a, PAX‐5, are BCL‐2 positive, and are negative for CD5, CD10, and BCL‐6. FAS mutations are frequently observed in PCMZL‐LPD and help distinguish these disorders from other extranodal marginal zone or MALT lymphomas [43]. The IGH/MALT1 t(14;18)(q32,q21) has been reported in up to 27% of PCMZL‐LPD [38]. Other translocations like IGH/BCL2t(3;14)(p14.1;q32), FOXP1/IGH, which can be seen in MALT lymphomas, have been rarely reported in PCMZL [44]. A high prevalence of hepatitis C virus (HCV) infection has been reported in patients with PCMZL‐LPD, sometimes associated with a peculiar subcutaneous lipoma‐like morphology [45]. Two immunogenetically distinct subtypes of PCMZL‐LPD have been identified, based on the immunoglobulin heavy chain IgH gene rearrangements. Most cases exhibit class‐switched immunoglobulins, predominantly IgG+, and are characterized by a prominent reactive T‐helper cell infiltrate and abundant plasma cells, features reminiscent of reactive lymphoid hyperplasia [46]. Neoplastic cells are often a minority, and they usually do not express IRTA1 and CXCR3. The class‐switched subtype represents the most indolent form of PCMZL‐LPD, is occasionally preceded by cutaneous lymphoid hyperplasia, and is associated with a lower risk of systemic dissemination [43]. The second subtype is nonclass switched (commonly IgM+) and has more MALT lymphoma‐like features. Neoplastic B‐cells are predominant, and they often express IRTA1 and CXCR3. MYD88 mutations are reported in some cases of nonclass‐switched IgM. This second subtype is more frequently associated with extracutaneous involvement and has a relatively more aggressive course, although prognosis remains excellent [22]. Although still subject to debate, some experts propose that the class‐switched form could be classified as a ‘lymphoproliferative disorder’ (LPD), whereas the nonclass‐switched subtype may represent a true but entirely indolent lymphoma [47].

### 3.3 Diagnosis and Staging

Diagnosis of PCMZL‐LPD requires an excisional biopsy of involved skin and the exclusion of noncutaneous disease [36]. However, in PCMZL‐LPD, the utility of routine bone marrow biopsy in staging remains controversial, and it is usually not recommended [18], except for patients with unexpected cytopenias or lymphocytosis. Studies demonstrated that such procedures are positive in 2%–23% of cases, and bone marrow involvement is barely the only extranodal involvement [48, 49, 50]. Current recommendations continue to support a comprehensive staging workup, which includes a thorough medical history and physical examination, complete blood count, comprehensive serum chemistry panel, and imaging studies such as chest/abdomen/pelvis CT [26]. The use of PET‐TC in this setting is controversial, and there is no formal recommendation, as FDG avidity in skin MZL is reported as low or moderate [51, 52]. A TNM staging is recommended [21, 22]. In endemic areas, *Borrelia Burgdorferi* testing (serology and DNA testing on lesional skin by PCR) should be recommended [43].

### 3.4 Treatment

PCMZL‐LPD has an indolent course and excellent prognosis, with a disease‐specific survival (DSS) of 99% [22]. Skin relapses are common (around 50%), while extracutaneous progression is rare. For all these reasons, treatment strategies prioritize minimally invasive and low‐morbidity approaches, tailored to lesion number, location, and symptomatology. Initial therapy for patients with solitary or localized lesions is local radiation therapy. Dose varied from 20 to 45 Gy [27]. The EORTC/ISCL recommends a dose range of 20 to 36 Gy for primary cutaneous marginal zone lymphoma. For symptomatic treatment of multifocal disease, low‐dose RT (2 × 2Gy) is often effective and nontoxic [53]. Surgical excision may be used for solitary lesions not suitable for radiation therapy. Patients with multifocal disease may be observed. Once symptomatic, lesions may be irradiated or surgically excised [22]. Systemic therapies, such as Rituximab, may be an option for patients with symptomatic, refractory, or generalized disease [18]. Antibiotic treatment may be considered for patients with *B. burgdorferi* ‐associated PCMZL‐LPD (generally consisting of cephalosporins +/− tetracyclines). However, discordant data exist about cutaneous MZL recession after antibiotic treatment and are based on case reports [22, 39]. In addition, in patients with PCMZL‐LPD associated with hepatitis C virus infection, antiviral therapy may provide clinical benefit in some cases [54, 55].

## 4 Primary Cutaneous Diffuse Large B‐Cell Lymphoma, Leg Type (PCDLBCL‐LT)

Diffuse Large B‐cell Lymphoma (DLBCL) is the most frequently diagnosed subtype of non‐Hodgkin lymphoma. It encompasses a broad and heterogeneous group of malignancies that differ significantly in their clinical presentation, histopathological features, genetic alterations, and treatment responses. Among the recognized subtypes of DLBCL, the leg‐type variant (PCDLBCL‐LT), represents a distinct clinical and pathologic entity. The recognition of PCDLBCL‐LT as a separate entity in the WHO–EORTC and ICC classifications underscores its clinical relevance and the need for tailored diagnostic and therapeutic approaches. Understanding the biological behavior of this lymphoma subtype is crucial for optimizing patient outcomes, as it generally requires more intensive treatment compared to indolent cutaneous B‐cell lymphomas [1, 2].

Reports have highlighted the existence of primary cutaneous large B‐cell lymphomas that do not fit neatly into the current dichotomy between PCFCL and PCDLBCL‐LT. These cases, sometimes referred to as PBCL NOS/unclassifiable, show morphological and clinical features that are intermediate between the indolent course of PCFCL and the aggressive behavior of PCDLBCL‐LT. Clinically, their prognosis also appears to be intermediate, with outcomes less favorable than typical PCFCL but more favorable than classic PCDLBCL‐LT. Although not included in the most recent WHO classifications, these cases highlight the heterogeneity of cutaneous large B‐cell lymphomas and may represent intermediate forms with overlapping features [56, 57, 58, 59].

### 4.1 Clinical Features

PCDLBCL‐LT primarily occurs in older adults, with a median age at diagnosis typically between the seventh and eighth decades. In contrast to most other lymphomas, this subtype shows a notable female predominance. Clinically, patients commonly present with one or more rapidly growing cutaneous tumors localized to the lower extremities. These lesions may appear as solitary nodules or as multiple, multifocal tumors. They are characteristically erythematous to violaceous in colour, have a firm to indurated consistency upon palpation (Figure 1E,F). Ulceration is common, particularly as the disease progresses. The aggressive nature of these lesions, combined with their unique localization and appearance, makes clinical recognition essential for prompt diagnosis and initiation of appropriate treatment [22]. In some instances, the cutaneous manifestations of PCDLBCL‐LT may closely resemble inflammatory or infectious dermatoses, including panniculitis, erythema induratum of Bazin or subcutaneous panniculitis‐like T cell lymphoma. This clinical overlap can lead to initial misdiagnosis, resulting in delays in appropriate diagnostic evaluation and initiation of definitive treatment. The erythematous hue, localized edema, and occasional tenderness of the lesions often mimic an infectious process, potentially reinforcing a misleading clinical impression. Therefore, in elderly patients presenting with persistent, atypical, or treatment‐refractory skin lesions—especially on the lower limbs—a high index of suspicion for cutaneous lymphoma is warranted to ensure timely recognition and management [60].

### 4.2 Histopathology and Biological Features

PCDLBCL‐LT is characterized by a diffuse, dense infiltrate of large atypical lymphoid cells predominantly involving the dermis and frequently extending into the subcutaneous tissue. The neoplastic cells are typically arranged in cohesive sheets and exhibit marked cytologic atypia, including round to oval vesicular nuclei, prominent central nucleoli, and abundant amphophilic to eosinophilic cytoplasm. A high mitotic rate and an elevated proliferative index, as demonstrated by Ki‐67 immunostaining often exceeding 70%, indicate the tumor's high grade and aggressive biological behavior. Immunophenotypic analysis demonstrates robust expression of pan‐B‐cell markers such as CD20, CD79a, and PAX5. The tumor cells also consistently express IgM, BCL2, and MUM1, while lacking CD10 expression. This immunoprofile is consistent with a nongerminal center B‐cell (non‐GCB) phenotype, which is associated with an unfavorable prognosis and reflects an activated B‐cell‐like molecular subtype. PCDLBCL‐LT is typically EBER‐negative [61]. BCL2 positivity serves as a critical diagnostic marker, aiding in the differentiation of PCDLBCL‐LT from other PBCL, particularly PCFCL, which typically lacks BCL2 and MUM1 expression. This differential expression reflects underlying biological differences and contributes to the more aggressive clinical behavior observed in PCDLBCL‐LT [19].

The immunophenotypic profile of PCDLBCL‐LT is not only diagnostically distinctive but also reflective of its biologically aggressive nature. Beyond the consistent expression of BCL2, a considerable proportion of cases demonstrate overexpression of c‐MYC, a transcriptional regulator implicated in cell cycle progression and oncogenesis. The adverse prognostic significance of double expressor (DE) status—defined by co‐expression of MYC and BCL2—and double‐hit/triple‐hit (DH/TH) status—characterized by translocations involving MYC and BCL2 and/or BCL6—is well established in nodal diffuse large B‐cell lymphomas. However, the prognostic relevance of DE and DH/TH status in primary cutaneous PCDLBCL remains inconclusive due to discordant observation [62, 63]. Similarly, the prognostic impact of MYC rearrangements is not completely understood [19].

Recent molecular studies have revealed the biological heterogeneity of PCDLBCL‐LT and identified recurrent genetic alterations driving its pathogenesis and aggressiveness. Activating mutations of MYD88 and mutations in CD79B are detected in a significant proportion of cases. These alterations play a central role in promoting constitutive activation of the NF‐κB signaling pathway, thereby supporting malignant B‐cell survival and proliferation. In addition to these oncogenic drivers, loss‐of‐function events involving tumor suppressor genes—most notably deletions or inactivation of *CDKN2A*—further contribute to deregulated cell cycle control and tumor progression [64, 65].

### 4.3 Diagnosis and Staging

In PCDLBCL‐LT, radiologic evaluation typically reveals nodular, poorly defined lesions localized within the subcutaneous tissue of the lower extremities. On PET‐CT with FDG imaging, these lesions exhibit pronounced FDG uptake, reflecting this aggressive lymphoma subtype's characteristic high proliferative and metabolic activity. Precise staging should be conducted using the EORTC/ISCL TNM classification system [66]. Risk stratification tools such as the International Prognostic Index (IPI) may be considered in the clinical evaluation of PCDLBCL‐LT; however, it is important to acknowledge that the IPI has not been specifically validated for this lymphoma subtype. While the IPI provides a general framework for assessing prognosis in systemic DLBCL, its applicability to cutaneous variants remains limited, and its prognostic accuracy in PCDLBCL‐LT should be interpreted with caution [67].

### 4.4 Treatment

Due to their frequent occurrence in elderly individuals—many of whom are over the age of 80—PCDLBCL‐LT has often been managed with radiotherapy alone or with palliative‐intent chemotherapy. This conservative therapeutic approach, influenced mainly by patient age and comorbidities, has been associated with relatively poor outcomes [68]. When PCDLBCL‐LT is localized to the leg, radiotherapy represents a valuable therapeutic modality. In patients with localized stages, particularly elderly individuals, radiotherapy alone may represent a suitable therapeutic option, with a recommended dose exceeding 40 Gy. The addition of localized radiotherapy to systemic treatment can enhance disease control and improve overall survival compared to systemic therapy alone [69]. In cases presenting with a solitary lesion or lesions confined to a single anatomical region, surgical excision may be considered with symptomatic intent [70]. Treatment with R‐CHOP in patients with PCDLBCL‐LT has demonstrated a complete response rate exceeding 90% and a 3‐year overall survival rate greater than 70%. Although derived from retrospective analyses, these outcomes—particularly when compared to historical cohorts treated with alternative, less intensive regimens—strongly indicate that the prognosis of this clinically aggressive and life‐threatening lymphoma can be significantly improved using age‐adapted rituximab‐based chemoimmunotherapy. Many PCDLBCL‐LT cases belong to the non‐GCB/ABC subtype, which is characterized by poorer response to chemotherapy and contributes to the unfavorable outcomes observed in this entity. These findings underscore the therapeutic potential of tailored systemic treatment even in elderly populations, where disease aggressiveness must be balanced against treatment tolerability [71]. Ibrutinib has shown encouraging results in patients with relapsed or refractory PCDLBCL‐LT, with documented durable remissions, even at low doses [72]. When used in combination with agents like R‐EPOCH, venetoclax, or lenalidomide, responses appear more robust and prolonged. Resistance mechanisms—such as CARD11 mutations and BCL2 amplification—underscore the importance of genomic monitoring and the potential advantage of tailored combination strategies [73, 74]. Currently, there are no robust clinical data specifically evaluating the efficacy of other innovative therapies—such as antibody–drug conjugates, bispecific antibodies, or chimeric antigen receptor T‐cell (CAR‐T) therapies—in the treatment of PCDLBCL‐LT. These advanced therapeutic modalities have shown promising results in relapsed/refractory systemic DLBCL, particularly in high‐risk or treatment‐resistant cases. However, their use in PCDLBCL‐LT remains largely unstudied, and clinical experience is limited to anecdotal reports or extrapolation from systemic disease settings.

## 5 Intravascular Large B‐Cell Lymphoma (IVLBCL)

Intravascular large B‐cell lymphoma (IVLBCL) is a very rare and aggressive form of B‐cell lymphoma that presents with cutaneous involvement in approximately 40% of cases. In nearly 30% of cases, the skin is the sole site of disease at onset (primary cutaneous IVLBCL). The clinical presentation is highly heterogeneous and may include painful indurated erythematous eruptions, poorly circumscribed violaceous plaques, lesions mimicking cellulitis, large solitary plaques, tumors, ulcerated nodules, among others [75]. In addition to cutaneous manifestations, this rare subtype is frequently associated with central nervous system involvement [76]. IVLBCL is often IgM‐positive and has robust expression of BCL2 and MUM1. The demonstration of clonal immunoglobulin gene rearrangements may serve as a useful diagnostic tool, given the peculiar presentation of this entity [77]. Targeted sequencing studies have revealed MYD88 L265P mutations in approximately 44% of cases, while CD79B mutations occur in around 26% of patients. MYC rearrangements are uncommon and not typically associated with this lymphoma subtype [78]. R‐CHOP‐based immunochemotherapy remains the standard first‐line regimen. IVLBCL is considered part of the spectrum of lymphomas arising in immune‐privileged sites, which explains its high propensity for central nervous system involvement; therefore, attention should be paid to CNS staging, including consideration of medicated lumbar puncture, and the use of high‐dose methotrexate may be recommended in some cases [76]. Patients with skin‐limited IVLBCL demonstrate a trend toward better overall survival compared to those with systemic involvement. MYD88 and CD79B mutations are observed in both cutaneous and systemic forms, and do not appear to correlate with clinical behavior or prognosis [79].

## 6 EBV‐Positive Mucocutaneous Ulcer (EBVMCU)

EBVMCU is classified as lymphoid proliferations and lymphomas associated with immune deficiency and dysregulation.

### 6.1 Clinical Features

EBVMCU is defined as a solitary, well‐circumscribed ulcerative lesion affecting the skin, oropharyngeal mucosa, or gastrointestinal tract, arising in the context of immunosuppression. This entity is typically observed in settings of immunosuppression, such as advanced age (immunosenescence), iatrogenic immunosuppression (e.g., posttransplant or autoimmune disease therapy), or primary immunodeficiency [2].

### 6.2 Histopathology and Biological Features

EBVMCU is characterized by a dense polymorphic inflammatory infiltrate in which large, transformed B cells resembling Hodgkin/Reed–Sternberg cells are a prominent component. These atypical B cells are consistently positive for Epstein–Barr virus–encoded RNA by in situ hybridization, confirming their EBV‐driven origin. The large neoplastic cells express PAX5 and demonstrate variable expression of CD20 on immunophenotypic analysis. They typically exhibit a non–germinal center B‐cell phenotype, with expression of markers such as IRF4/MUM1, CD10 (infrequent), and BCL6. CD30 expression is almost universal, and approximately 50% of cases also coexpress CD15, further mimicking classical Hodgkin lymphoma. Despite these aggressive morphologic and immunophenotypic features, EBVMCU usually follows an indolent clinical course and may regress spontaneously or with reduction or cessation of immunosuppressive therapy [80].

EBVMCU typically presents as a localized process without evidence of systemic dissemination, in contrast to aggressive EBV‐associated lymphomas. This distinct clinical behavior underscores the importance of accurate recognition and diagnosis, as misclassification may lead to unnecessary and potentially harmful overtreatment with intensive chemotherapy [81].

### 6.3 Treatment

The primary approach to managing EBVMCU is conservative, with initial treatment focused on reducing or discontinuing immunosuppressive therapy that may be compromising immune surveillance. In many cases, this intervention alone is sufficient to induce lesion regression, reflecting the self‐limited nature of the disease when immune function is restored. Close clinical monitoring is essential, and additional therapies are generally reserved for refractory or progressive diseases [82]. Most patients with EBVMCU achieve complete remission following reduction or discontinuation of immunosuppressive therapy, with follow‐up studies confirming ulcer resolution and a low likelihood of progression to systemic lymphoma. This favorable outcome underscores the indolent nature of EBVMCU when immune competence is restored. However, in rare cases, persistent or nonresolving EBVMCU has been reported to transform into EBV‐positive DLBCL, highlighting the need for careful long‐term monitoring, particularly in patients with ongoing immunosuppression or atypical clinical courses [83]. Rituximab has been effectively employed in refractory cases of EBVMCU, achieving complete remission in several reports. Its use is particularly valuable in patients for whom significant reduction of immunosuppressive therapy is not feasible, such as those with underlying autoimmune diseases or solid organ transplants, where maintaining immunosuppression is critical. Additionally, local external beam radiotherapy, administered at 20 to 45 Gy doses, has demonstrated high clinical response rates with a favorable toxicity profile. Radiotherapy is especially advantageous in scenarios requiring rapid lesion control, such as symptomatic ulcers or lesions at anatomically sensitive sites [84]. In summary, EBVMCU is a benign and self‐limiting lymphoproliferative disorder that exemplifies the complex interplay between viral oncogenesis and host immune regulation. Recognition of this distinct clinical and pathological entity carries significant therapeutic implications, as accurate diagnosis can prevent overtreatment with cytotoxic regimens and support a conservative management approach. Moreover, appropriate identification allows clinicians to reassure patients regarding the typically excellent prognosis and low risk of systemic progression associated with EBVMCU [85].

## 7 Conclusion

PCBCLs represent a biologically and clinically heterogeneous group of lymphoproliferative disorders that require careful diagnostic distinction and tailored management strategies (Table 3). Despite significant progress in the understanding of their histopathologic, immunophenotypic, and molecular characteristics, along with the recognition of rare entities such as EBVMCU, several diagnostic and therapeutic challenges remain unresolved. Key controversies include the distinction between reactive and neoplastic infiltrates, the delineation between PCFCL and PCDLBCL, and the prognostic subcategorization of aggressive subtypes. Furthermore, data on the use of innovative therapies such as antibody–drug conjugates, bispecific antibodies, and CAR‐T cell therapies in PCBCL are currently lacking. Looking ahead, the identification of novel genetic markers and the clinical application of emerging therapeutic agents hold promise for improving diagnostic precision and expanding treatment options, ultimately enhancing patient outcomes in both indolent and aggressive forms of PCBCL.

**TABLE 3:** Management of cutaneous B‐cell lymphomas.

	PCFCL	PCMZL	PCDLBCL‐LT	IVLBCL	EBVMCU
Therapy					
Localized disease	ISRT or surgical excision or IL steroids or IL rituximab	ISRT or surgical excision or IL steroids or IL rituximab	ISRT or rituximab + anthracycline‐based chemotherapy +/− ISRT	—	Spontaneous resolution after reduction of immunosuppressive agents
Generalized disease (skin only)	Intravenous rituximab + − ISRT or surgical excision	W&W, ISRT or surgical excision +/− Intravenous rituximab	Rituximab + anthracycline‐based chemotherapy +/− ISRT	—	—
Extracutaneous disease	Manage as Follicular Lymphoma	Manage as Nodal Marginal Lymphoma	Manage as DLBCL	Manage as DLBCL with addition of CNS oriented therapy	—
Prognosis	5‐y OS 95%	5‐y OS > 95%	5‐y OS 50%	5‐y OS 26%–73%	Excellent
Relapse rate	20%–50%	50%	65%	Significant	< 3%

Abbreviations: CNS, central nervous system; IL, intralesional; ISRT, involved‐site radiotherapy; OS, overall survival; W&W, watch and wait.

## Author Contributions

A.B., E.C., B.B. wrote the first draft of the manuscript. A.B., F.B. and C.V. critically reviewed and edited the manuscript. A.B. and C.V. conceptualized the manuscript. All authors have read and agreed to the final version of the manuscript.

## Conflicts of Interest

The authors declare no conflicts of interest.

## Data Availability

Data sharing not applicable to this article as no datasets were generated or analyzed during the current study.
